# Overcoming sorafenib evasion in hepatocellular carcinoma using CXCR4-targeted nanoparticles to co-deliver MEK-inhibitors

**DOI:** 10.1038/srep44123

**Published:** 2017-03-09

**Authors:** Yunching Chen, Ya-Chi Liu, Yun-Chieh Sung, Rakesh R. Ramjiawan, Ts-Ting Lin, Chih-Chun Chang, Kuo-Shyang Jeng, Chiung-Fang Chang, Chun-Hung Liu, Dong-Yu Gao, Fu-Fei Hsu, Annique M. Duyverman, Shuji Kitahara, Peigen Huang, Simona Dima, Irinel Popescu, Keith T. Flaherty, Andrew X. Zhu, Nabeel Bardeesy, Rakesh K. Jain, Cyril H. Benes, Dan G. Duda

**Affiliations:** 1 Steele Laboratories for Tumor Biology, Department of Radiation Oncology, Massachusetts General Hospital and Harvard Medical School, Boston, USA; 2 Institute of Biomedical Engineering, National Tsing Hua University, Hsinchu, Taiwan; 3 Angiogenesis Laboratory, Department of Medical Oncology, VU University Medical Center, Amsterdam, The Netherlands; 4 Department of Surgery, Far Eastern Memorial Hospital, New Taipei City, Taiwan; 5 Institute of Biomedical Sciences, Academia Sinica, Taipei, Taiwan; 6 University Medical Center, Utrecht, The Netherlands; 7 Dan Setlacec Center of General Surgery and Liver Transplantation, Fundeni Clinical Institute, Bucharest, Romania; 8 Department of Medicine, Massachusetts General Hospital and Harvard Medical School, Boston, USA

## Abstract

Sorafenib is a RAF inhibitor approved for several cancers, including hepatocellular carcinoma (HCC). Inhibition of RAF kinases can induce a dose-dependent “paradoxical” upregulation of the downstream mitogen-activated protein kinase (MAPK) pathway in cancer cells. It is unknown whether “paradoxical” ERK activation occurs after sorafenib therapy in HCC, and if so, if it impacts the therapeutic efficacy. Here, we demonstrate that RAF inhibition by sorafenib rapidly leads to RAF dimerization and ERK activation in HCCs, which contributes to treatment evasion. The transactivation of RAF dimers and ERK signaling promotes HCC cell survival, prevents apoptosis via downregulation of BIM and achieves immunosuppression by MAPK/NF-kB-dependent activation of PD-L1 gene expression. To overcome treatment evasion and reduce systemic effects, we developed CXCR4-targeted nanoparticles to co-deliver sorafenib with the MEK inhibitor AZD6244 in HCC. Using this approach, we preferentially and efficiently inactivated RAF/ERK, upregulated BIM and down-regulated PD-L1 expression in HCC, and facilitated intra-tumoral infiltration of cytotoxic CD8+ T cells. These effects resulted in a profound delay in tumor growth. Thus, this nano-delivery strategy to selectively target tumors and prevent the paradoxical ERK activation could increase the feasibility of dual RAF/MEK inhibition to overcome sorafenib treatment escape in HCC.

The efficacy of targeted therapy with kinase inhibitors in cancer is often limited by rapid treatment evasion. Treatment resistance may develop either due to additional mutations, by alternate mode of activation of the same pathway or alternative oncogenic pathways, or by dynamic reprogramming of the kinome^1^,^2^,^3^. One such mechanism is the “paradoxical” activation of MAP kinase (MAPK) pathway (RAF/MEK/ERK) by RAF inhibitors leading to adverse effects^4^. The use of RAF inhibitors such as vemurafenib or sorafenib in *BRAF*-wild-type cancers has been shown to lead to “paradoxical” activation of ERK in cutaneous squamous cell carcinoma, lung cancer or melanoma, likely due to a direct effect of the drug on RAF dimerization^2^,^4^,^5^.

Of the RAF inhibitors, the most widely used drug is sorafenib. Sorafenib is the standard therapy for advanced hepatocellular carcinoma (HCC) worldwide, and is also approved for advanced renal and thyroid cancers^6^,^7^. Sorafenib inhibits VEGFR and PDGFR tyrosine kinases, which is thought to exert anti-angiogenic and anti-fibrotic effects in HCC. We have previously shown that these effects are thwarted by treatment-induced hypoxia, which leads to CXCR4 upregulation in HCC and stromal cells and mediates metastasis progression and PD-L1-mediated immunosuppression^8^,^9^. On the other hand, sorafenib was developed as a cytotoxic agent to inhibit RAF kinases and downstream mitogen-activated protein kinases (MAPKs)^10^,^11^. However, HCCs only rarely harbor somatic mutation of the MAPK pathway such as *KRAS* or *BRAF*^V600E^ activating mutations^12^. This prompted us to examine whether paradoxical activation of MAPK pathway occurs after sorafenib treatment in HCC. The RAF/MEK/ERK signal transduction cascade is widely considered promote tumor progression. RAF/MEK/ERK pathway can regulate the activity and expression of members of the BCL-2 protein family to promote cell survival^13^. In addition, activation of RAF/MEK/ERK signaling may directly upregulate PD-L1 expression and promote an immunosuppressive tumor microenvironment^14^,^15^.

Recent efficacy studies and FDA-approval for a dual RAF/MEK inhibition approach in *BRAF*-mutant melanoma is supporting the clinical relevance of this escape mechanism, but significant toxicity concerns remain^16^,^17^. The importance of paradoxical activation in limiting therapeutic efficacy in *RAF*-wild type cancers remains unknown. Here, we examined the relevance of ERK activation in HCCs treated with sorafenib (the only approved systemic therapy for this disease), and developed a safer nanoparticle-based multi-drug delivery system to overcome treatment resistance.

## Results

### Sorafenib induces RAF heterodimerization and ERK activation in *BRAF*-wild type HCC cells *in vitro* and *in vivo*

**Figure 1:**
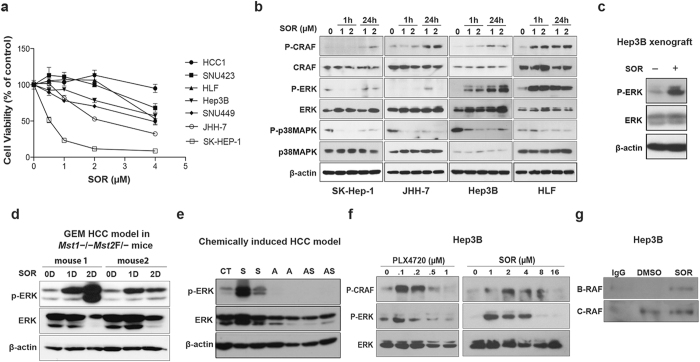
Paradoxical activation of ERK occurs after sorafenib treatment in *BRAF*^WT^ HCC cells. (**a**) *In vitro* sensitivity of human and murine HCC cells to clinically relevant doses of sorafenib: The IC_50_ values indicate that JHH-7 and SK-Hep-1 cells are more sensitive (2.26 μM and 0.5 μM, respectively), while most human HCC cell lines are quite resistant (IC_50_ of 6.4 μM for SNU-423 cells; 4.75 μM for HLF cells; 4.70 μM for Hep3B cells; 3.82 μM for SNU-449 cells). Similarly, murine HCC1 cells are resistant to sorafenib at these doses (n = 6). (**b**) Rapid CRAF and ERK activation in *BRAF*^WT^ HCC cells but not in *BRAF*^V600^ HCC cells (SK-Hep-1) after sorafenib treatment. All human and murine HCC cell lines tested showed down-regulation of p38MAPK activity after sorafenib treatment. (**c**) Sorafenib treatment increased ERK activity in orthotopic xenograft HCCs. (**d**) Spontaneously arising HCCs in *Mst1*^−/−^*Mst2*^Flox/-^ transgenic mice (**e**) and chemically induced HCCs in mice treated with CCl_4_ for 28 weeks. (**f**) CRAF and ERK activation is rapid in Hep3B cells after exposure to PLX4720 and sorafenib. (**g**) Transactivation of RAF dimers occurred in Hep3B cells (HCC-1 cells) treated with 2 uM sorafenib for 1 h.

We found a moderate and variable cytotoxicity after sorafenib treatment in a panel of HCC cell lines, consistent with previous studies and with the transient and moderate responses typically seen in HCC patients^18^,^19^. The *BRAF*-wild type HCC cells showed differential sensitivity, with IC_50_ values ranging from 2 μM (a clinically relevant concentration^18^) to over 5 μM (Fig. 1a). Of note, *BRAF*^V600^ mutant HCC cells (SK-Hep-1) were more sensitive to sorafenib (IC_50_ = 0.5 μM). We first evaluated the effects of sorafenib on RAF/ERK and MAP kinase p38 (p38MAPK) activation — two relevant targets of this drug in HCC cells^20^,^21^. As expected, sorafenib efficiently inhibited p38MAPK activity in all cell lines (Fig. 1b and Table S1). However, all *BRAF*-wild type HCC cell lines tested showed undetectable to low levels of CRAF and ERK phosphorylation at baseline (Fig. 1b). Moreover, sorafenib treatment induced a rapid and sustained increase in ERK activation in *BRAF*-wild type but not in *BRAF*^V600E^ mutant HCC cells (Fig. 1b and Table S1). The robust ERK1/2 phosphorylation was more substantial in sorafenib-resistant HCC cell lines (Fig. 1a,b). Similar to the ERK activation seen in HCC cells *in vitro*, we found that sorafenib increased ERK activation in orthotopic Hep3B xenografts and in two murine models of spontaneous HCC (in *Mst1*^−/−^*Mst2*^F/−^ mice and chemically-induced carcinogenesis) (Fig. 1c–e)^22^,^23^.

To test whether this effect is due to inefficient BRAF inhibition by sorafenib, we repeated the experiment using supra-physiological concentrations of sorafenib (4–16 μM)^24^. At these high concentrations, sorafenib inhibited CRAF and ERK activity similarly to PLX4720—a potent and specific BRAF inhibitor—in *BRAF*-wild type HCC cells (Fig. 1f). We also examined if ERK activation was due to RAF heterodimerization in *BRAF*-wild type HCC cells—as previously shown with BRAF inhibitors in other cancers^4^. Indeed, after treating HCC cells with sorafenib and evaluating the presence of BRAF in CRAF immunoprecipitate, we found that BRAF heterodimerized with CRAF (Figs 1g and S1).

### Paradoxical activation of ERK promotes resistance to sorafenib via degradation of Bim in *BRAF*-wild type HCC cells

**Figure 2:**
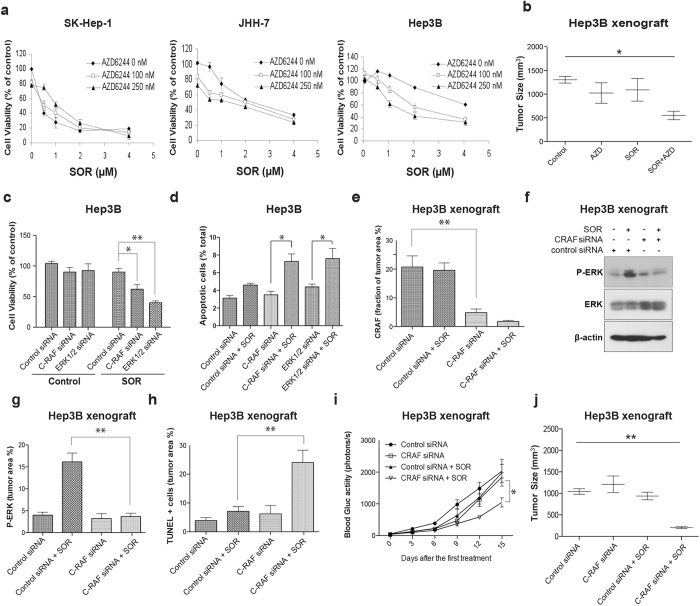
Paradoxical activation of ERK promotes sorafenib resistance in *BRAF*^WT^ HCC cells. (**a**) Effect of MEK inhibition on cell sensitivity to sorafenib: Sorafenib and AZD6244 show comparable cytotoxicity and no additive effects against SK-Hep-1 *BRAF*^V600^ mutant HCC cells. Inhibition of ERK activity with a pharmacologic MEK inhibitor (AZD6244) renders Hep3B and HLF cells sensitive to sorafenib. (n = 6). (**b**) Treatment with combination of sorafenib and AZD6244 resulted in synergistic tumor growth delay (n = 4). (**c**) Combination of sorafenib with CRAF or ERK siRNA leads to a synergistic cytotoxic effect in Hep3B cells. (**d**) Combination of sorafenib with CRAF or ERK siRNA increases apoptosis in Hep3B cells. Combination of sorafenib with CRAF siRNA encapsulated in liposome-based nanoparticles silenced CRAF expression (**e**), downregulated ERK activation (**f**,**g**) and increased cell apoptosis (**h**) in orthotopic Hep3B xenografts in nude mice, which resulted in synergistic tumor growth delay, as estimated by blood Gluc measurements (**i**) and tumor size (**j**). The data are the mean value ± S.E.M., *p < 0.05, **p < 0.01, ***p < 0.001.

Next, we determined whether the increased ERK phosphorylation after sorafenib treatment mediates HCC cell viability and tumor growth. We found that blocking ERK activation with AZD6244 significantly reduced HCC cell viability both *in vitro* and *in vivo* when combined with sorafenib treatment (Fig. 2a,b). Furthermore, we examined whether knocking down CRAF or ERK expression—and thus preventing sorafenib-induced RAF dimer transactivation and consequent ERK activation—could also affect *viability* of *BRAF*^WT^ HCC. Downregulation of CRAF alone did not affect HCC cell viability or primary tumor growth (Fig. 2c,d). However, when siRNA-induced CRAF knockdown was combined with sorafenib, the knock down of CRAF reduced ERK activation, decreased HCC cell viability, triggered apoptosis and caused a significant delay in HCC growth (Figs 2c–j and S2).

**Figure 3:**
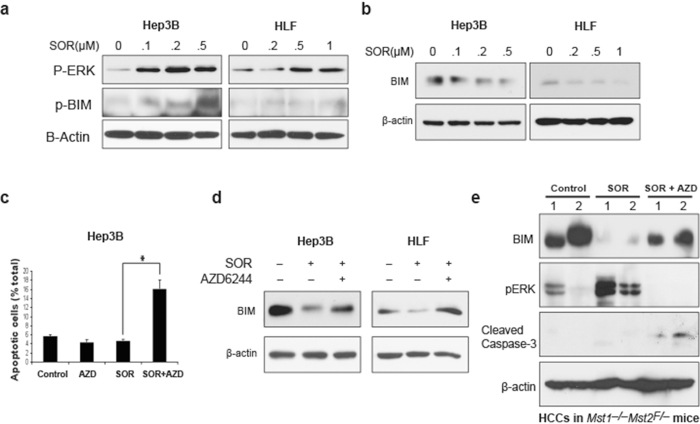
Resistance of sorafenib in *BRAF*^WT^ HCC cells is due to degradation of Bim. (**a**) Bim phosphorylation in BRAF^WT^ HCC cells 24 hours after BRAF inhibition with sorafenib. (**b**) Bim degradation in BRAF^WT^ HCC cells 48 hours after BRAF inhibition with sorafenib. Inhibition of ERK increases apoptosis (**c**) and decreases Bim degradation after sorafenib treatment in *BRAF*^WT^ HCC cells *in vitro* (**d**) and in spontaneously arising HCCs in *Mst1*^−/−^*Mst2*^Flox/−^ mice *in vivo* (**e**).

We further dissected how paradoxical ERK activation modulates HCC cell apoptosis. ERK mediates ubiquitination and degradation of Bim—a pro-apoptosis molecule—leading to resistance to chemotherapeutic drugs^25^,^26^. We found that sorafenib treatment alone led to Bim phosphorylation and degradation (Fig. 3a,b). However, Bim degradation was prevented when the HCC cells were treated with sorafenib in combination with AZD6244 both *in vitro* and *in vivo*, leading to apoptosis in HCC cells (Fig. 3c–e). Collectively, these data show that ERK activation and Bim degradation may mediate the rapidly acquired resistance to sorafenib in *BRAF*-wild type HCC.

### Co-delivery of sorafenib and the MEK inhibitor AZD6244 by the tumor-targeted nanoparticles prevents the paradoxical activation of ERK and PD-L1 expression and facilitates intra-tumoral infiltration of cytotoxic CD8+ T cells in HCC, resulting in enhanced anti-tumor efficacy

**Figure 4:**
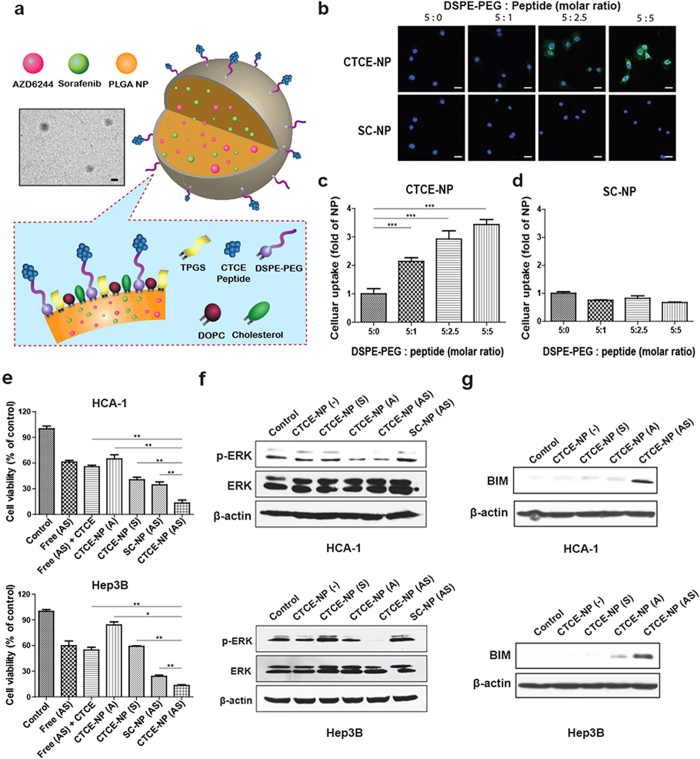
The NPs modified with CTCE peptides enhanced cellular uptake in HCC cells, exerted potent cytotoxic effects and prevented the paradoxical activation of ERK when loaded with sorafenib and the MEK inhibitor AZD6244. (**a**) Structures proposed for the CTCE-NPs with a representative TEM image (Scale bar = 100 nm). (**b**–**d**) Murine HCC cells (HCA-1 cells) were treated with C6-loaded NPs modified with CTCE peptides (CTCE-NPs) or scramble peptides (SC-NPs) at various ratio of DSPE-PEG/peptides for 4 hr. The cellular uptake of NPs was imaged and quantified with a Zeiss LSM 780 confocal microscope. (**e**) The cytotoxicity of sorafenib or AZD6244 (1 μM) in different formulations to HCC cells was measured using the MTT assay 48 hours after drug exposure (n = 4–6). (**f**) CTCE-NPs co-delivering sorafenib and AZD6244 prevented the effect of sorafenib on paradoxical activation of ERK in HCC cells. (**g**) CTCE-NPs loaded with sorafenib and the AZD6244 (0.25 μM) upregulated expression of Bim in HCC cells 24 hours after drug exposure. Scale bar = 50 μm. Free (AS), free-from AZD6244 and sorafenib; CTCE-NP (−), empty NPs modified with CTCE peptides; CTCE-NP (A), CTCE-NPs loaded with AZD6244; CTCE-NP (S), CTCE-NPs loaded with sorafenib; SC-NP (AS), AZD6244 and sorafenib loaded in NPs modified with scramble peptides.

To overcome this evasion mechanism and reduce systemic toxicities, we developed tumor-targeted nanoparticles (TTNPs), with the structure shown in Fig. 4a, to co-deliver sorafenib with a MEK inhibitor into HCC. To this end, we loaded sorafenib and the MEK inhibitor AZD6244 into NPs developed as previously described with several modifications^27^,^28^. The average diameters of drug-loaded TTNPs determined by DLS were 139.7 ± 9.7 nm, with poly-dispersity indexes (PDIs) of 0.425 ± 0.057. The efficacy of sorafenib and AZD6244 encapsulation in the NPs was approximately 70%. To more selectively target HCC, CTCE-9908, a peptide antagonist for CXCR4, was conjugated to NPs as a targeting ligand (CTCE-NPs)^29^,^30^. We examined the cellular uptake of CTCE-NPs containing a tracer molecule C6 using both murine HCC (HCA-1) and human HCC (Mahlavu and Hep3B) cell lines. As shown in Fig. 4b–d, the uptake of C6 was greater in HCC cells treated with targeted CTCE-NPs than in cells treated with non-targeted NPs modified with scramble peptides (SC-NPs) (Fig. S3). The uptake of CTCE-NPs was competitively inhibited by addition of free CTCE-9908 peptides in a dose-dependent manner (Fig. S3), indicating that the cellular uptake was ligand dependent. Furthermore, CTCE-NPs loaded with sorafenib and AZD6244 exerted more potent cytotoxic effects on HCC cells than the combination of the free agents, unloaded CTCE-NPs, loaded SC-NPs or CTCE-NPs loaded with each agent alone (Figs 4e and S4). In addition, co-delivery of sorafenib and AZD6244 by CTCE-NPs prevented the paradoxical activation of ERK and increased the expression of Bim (Figs 4f,g and S4). These results demonstrate a synergistic cell-killing effect when using CTCE-NPs loaded with sorafenib and AZD6244.

**Figure 5:**
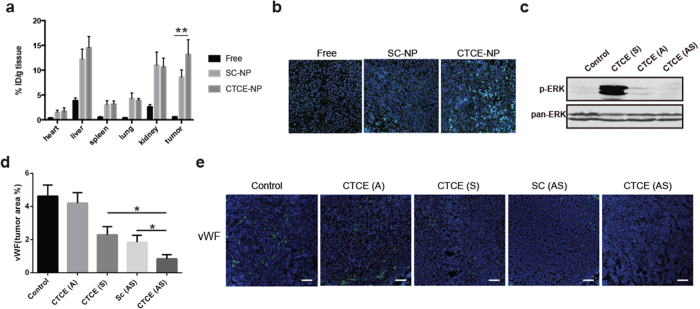
CTCE-NPs loaded with sorafenib and the MEK inhibitor AZD6244 suppressed angiogenesis in HCC. (**a**) The tissue distribution of C6 in different formulations was measured by a plate reader at excitation wavelength 485 nm and emission wavelength 538 nm (n = 7–10). (**b**) The intracellular tumor uptake of C6 in different formulations 4 h after intravenous administration was imaged by a confocal microscopy. (**c**) CTCE-NPs containing sorafenib and AZD6244 significantly decreased activation of ERK induced by sorafenib treatment. (**d**,**e**) Sorafenib and AZD6244 co-delivered by CTCE-NPs significantly reduced the mean vessel density in HCA-1 tumors of orthotopic HCC-bearing mice (n = 6–12). Data are representative of at least two independent experiments and mean values and S.E.M. are presented. *p < 0.05, **p < 0.01, ***p < 0.001.

Next, we evaluated the *in vivo* effects of CTCE-NPs loaded with sorafenib and AZD6244 on ERK activation as well as on tumor environment in orthotopic murine HCC models. As shown in Fig. 5a, increased tumor uptake of CTCE-NPs or non-targeted SC-NPs was observed compared with that of free drugs due to the EPR (enhanced permeability and retention) effect. Moreover, the enhanced intracellular uptake of CTCE-NPs was observed, indicating that NPs presenting a CXCR4 antagonist CTCE9908 peptide can increase tumor uptake in the murine HCC model (Figs 5b and S5). Furthermore, co-delivery of sorafenib and AZD6244 using CTCE-NPs significantly prevented ERK activation induced by sorafenib (Figs 5c and S6). Of note, this effect was achieved while maintaining a potent anti-angiogenic effect of sorafenib, as demonstrated by the decrease in tumor microvascular density (MVD) (Fig. 5d,e).

**Figure 6:**
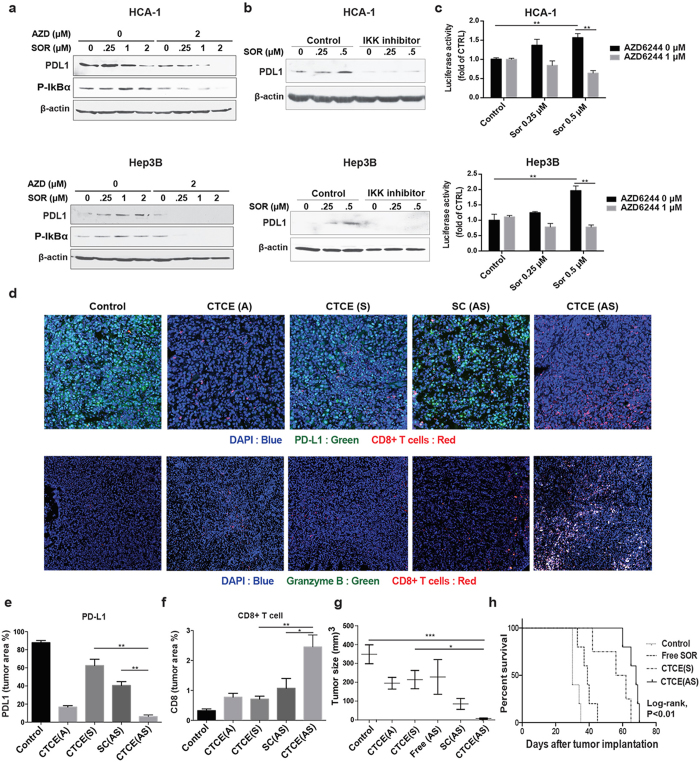
CTCE-NPs loaded with sorafenib and the MEK inhibitor AZD6244 suppressed PD-L1 expression and increased CD8 T cell accumulation in HCC. (**a**) Treatment of sorafenib at low doses increased the expression of PD-L1 and the activation of p-IκBα in HCCs, consistent with their paradoxical activation of ERK, while inhibition of MEK with AZD6244 prevented the effects of sorafenib. (**b**) Inhibition of NF-κB activation prevented the effects of sorafenib-induced PD-L1 expression. Changes of PD-L1 expression were detected 24 hours after drug exposure. (**c**) Sorafenib treatment increased NF-kB-driven luciferase activity in HCC cells. Combination of sorafenib and AZD6244 suppressed the effect of sorafenib-induced NF-kB activation. (**d**) Immunofluorescence staining of frozen HCA1 tumors (DAPI, blue; PD-L1 or Granzyme B, green; CD8+ T cells, red). (**e**,**f**) Quantification of PD-L1 expression and tumor infiltration with CD8+ cells performed in randomly selected fields within the HCA-1 tumors showed sorafenib and AZD6244 co-delivered by CTCE-NPs suppressed PD-L1 expression and increased the number of infiltrating CD8+ cells in HCA-1 tumors as compared to other treatment groups. (n = 5–7). (**g**) Tumor sizes in orthotopic tumor-bearing mice were significantly reduced as the result of treatment with CTCE NPs loaded with sorafenib and AZD6244 compared with other treatments (n = 4–10). (**h**) Overall survival was significantly prolonged in orthotopic HCC-bearing mice treated with sorafenib and AZD6244 loaded CTCE-NPs. Data are representative of at least two independent experiments and mean values and S.E.M. are presented. N represents number of mice in each treatment group. *p < 0.05, **p < 0.01, ***p < 0.001.

Finally, we evaluated the treatment-induced changes in programmed death-1-ligand 1 (PD-L1) expression, an immune checkpoint which could suppress cytotoxic CD8+ T cell proliferation and activation, resulting in immunosuppression and treatment resistance^31^. We examined surgical specimens of HCC from patients with recurring tumors after sorafenib treatment and observed profound ERK activation and increased PD-L1 expression in recurrent HCC (Figs S7 and S8). We further found that sorafenib treatment directly induced PD-L1 expression via ERK-mediated NF-kB activation in murine and human HCC cells in a dose dependent manner (at low doses), which was prevented by MEK or NF-kB inhibition (Figs 6a–c and S9). Moreover, co-delivery of sorafenib and AZD6244 using CTCE-NPs significantly reduced PD-L1 expression and increased cytotoxic T cell accumulation and activation in HCC *in vivo* (Fig. 6d–f). Treatment with CTCE-NPs loaded with sorafenib and AZD6244 induced potent tumor regression and increased overall survival in orthotopic HCC model in immunocompetent mice (Fig. 6g,h). Furthermore, the preclinical safety study indicated that the serum levels of hepatotoxicity makers such as aspartate aminotransferase (AST), alanine aminotransferase (ALT), alkaline phosphatase (ALP) and γ-Glutamyltransferase (γ-GT) remained the same as the untreated control animals after treatment of sorafenib and MEK inhibitors in CTCE-NPs (Table S2). We also examined the histology of the various organs of mice treated with sorafenib and AZD6244 in CTCE-NP by H&E staining. We detected no histological changes in the lungs, liver, spleen, kidneys or heart of C3H mice 24 hr after treatment (Fig. S10).

**Figure 7:**
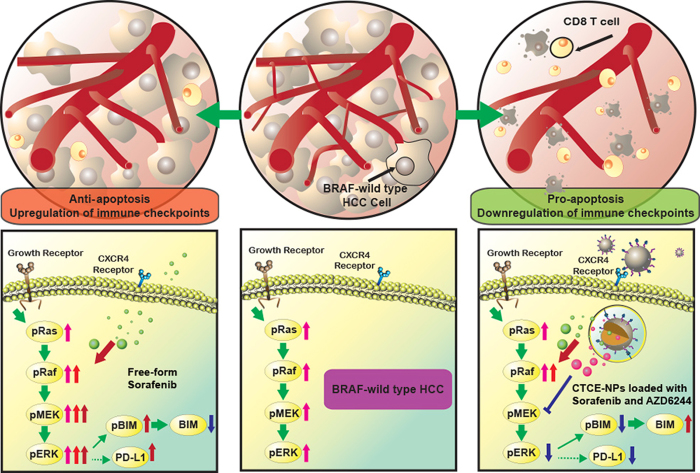
Schematic illustration of the mechanisms by which sorafenib and AZD6244-loaded CTCE-NPs overcome sorafenib treatment escape in HCC. Sorafenib transactivates RAF dimers and ERK signalling and results in BIM downregulation and PD-L1 upregulation, leading to resistance to sorafenib treatment in HCC. NPs modified with CTCE-9908 co-deliver sorafenib with the MEK inhibitor AZD6244 in HCC, downregulate RAF/ERK and PD-L1 expression, and facilitate intra-tumoral infiltration of cytotoxic CD8+ T cells, resulting in a profound delay in tumor growth.

In summary, we demonstrate that rapid ERK activation is a mediator of sorafenib resistance in HCC (Fig. 7). Co-delivery of sorafenib and MEK inhibitors via CXCR4-targeted NPs overcomes the cell autonomous mechanism of resistance to sorafenib in HCC as well as inhibits angiogenesis and converts the immunosuppressive microenvironment to an immunostimulatory microenvironment.

## Discussion

Multiple targeted therapies that entered phase III clinical trials in HCC failed to demonstrate superiority or non-inferiority to sorafenib. Thus, sorafenib remains standard of care for patients with advanced stage HCC. The poor understanding of sorafenib’s cell-autonomous mechanisms of action in HCC prompted us to examine whether the effect of sorafenib on HCC cell viability is dependent on RAF inhibition. Recently, the use of RAF inhibitors such as vemurafenib or sorafenib has been shown to paradoxically activate MAPK pathway in *BRAF-*wild type lung cancer or melanoma, due to induction of RAF (BRAF or CRAF) dimerization^2^,^4^,^32^. The clinical responses observed in patients with HCC patients (i.e., a transient delay in tumor growth followed by progression) is consistent with the moderate and highly variable cytotoxicity of sorafenib that we observed in various murine and human HCC cell lines. It suggests that inhibition of non-mutated cell-proliferation pathways (i.e., *BRAF-*wild type) may result in compensatory activation of downstream or other pathways that might become the rescue pathway for cell survival. Our data show RAF inhibition with knocking down BRAF/CRAF or using RAF inhibitors only moderately affected cell viability in *BRAF-*wild type HCCs. Moreover, partial inhibition of RAF using low doses of sorafenib or the specific *BRAF* inhibitor PLX4720 resulted in rapid MAPK activation due to *BRAF* heterodimerization as a cell-autonomous mechanism of resistance to sorafenib in *BRAF-*wild type HCCs.

We further studied how ERK activation mediates HCC cell survival by dissecting the signaling downstream of MAPK pathway. Bim is a member of the Bcl-2 homology 3 (BH3)-only subgroup of the Bcl-2 family. Bim induces apoptosis by blocking the activity of the anti-apoptotic members of the Bcl-2 family. It has been previously shown that ERK mediates ubiquitination and degradation of Bim through the Bim phosphorylation, which leads to resistance to chemotherapeutic drugs^25^,^26^. We show that the rapid activation of the MAPK pathway – induced by sorafenib treatment – leads to Bim degradation and protection from apoptosis in HCC cells. When CRAF knockdown or MEK inhibition—mediated by CRAF siRNAs or MEK blockade—was combined with sorafenib, we found reduced ERK activation, increased Bim and apoptosis induction and thus decreased HCC cell viability.

Next, we evaluated whether the sorafenib-induced paradoxical activation of ERK contributed to the changes of PD-L1 expression in HCC. We have previously shown that sorafenib increases PD-L1 expression in HCC *in vivo* and others have linked PD-L1 upregulation with MAPK activation in melanomas resistant to BRAF inhibition^8^,^33^. In this study, we observed the increased PD-L1 expression in HCC from patients with recurring tumors after sorafenib treatment. *BRAF-*wild type HCC cells showed enhanced ERK activation and PD-L1 expression while treated with sorafenib. The increased PD-L1 expression is transcriptionally modulated by NF-kB. MEK inhibition counteracts sorafenib resistance associated with MAPK activation and PD-L1-mediated immunosuppression. Combination of sorafenib and MEK inhibitors revealed dual therapeutic effects with apoptosis induction and reduction in PD-L1 expression, leading to increased accumulation and activation of tumor-infiltrating cytotoxic T cells. The targeted therapy in combination with immunotherapy not only overcomes resistance, but also provides long-term clinical benefit in HCC patients.

To overcome this evasion mechanism and reduce systemic toxicities, we developed CXCR4-targeted nanoparticles to co-deliver sorafenib with the MEK inhibitor AZD6244 into HCC. We have previously demonstrated that CXCR4 antagonists on the surface of targeted NPs display dual functions, serving both as tumor-targeting ligands and as inhibitors of CXCR4/SDF1α to suppress the accumulation and activation of stromal cells with tumor-promoting properties^28^. In this study, we utilized a peptide inhibitor of CXCR4—CTCE-9908 peptide to modified NPs. The multifunctional NPs loaded with sorafenib and MEK inhibitor achieve potent MAPK inhibition, induced significantly increased cell apoptosis, suppressed PD-L1 expression and inhibited immunosuppression in HCC.

These findings were consistent among various human and murine HCC cells *in vitro*, and were reproduced *in vivo* using both orthotopically implanted and chemically-induced murine HCC tumors. Molecular profiling of human HCCs has shown that that most tumors harbor no RAF-activating mutations and are indeed *BRAF-*wild type^34^. Moreover, consistent with our *in vitro* data obtained from human HCC cell lines, sorafenib treatment activates the RAF/ERK pathway and increases PD-L1 expression in patients with HCC.

Collectively, these data demonstrate that sorafenib can cause rapid RAF dimerization and ERK activation, leading to resistance to sorafenib in *BRAF-*wild type HCC cells. Adding a MEK inhibitor to sorafenib is clinically feasible^35^, but this strategy may be limited by the specific pharmacokinetics of these agents, and equally important by adverse systemic effects. We addressed this challenge by using a CXCR4-targeted nano-delivery strategy to target HCCs and co-deliver sorafenib with a MEK inhibitor. We showed that treatment with sorafenib and MEK inhibitors co-delivered by tumor-targeted NPs PEG-PLGA revealed more potent tumor growth inhibition effect when compared to the free-form drug cocktail without causing unwanted toxicity, indicating the CTCE-NP formulation could increase the therapeutic effects and therapeutic window of combined sorafenib and MEK inhibitors. This approach may be useful in safely preventing a key cell autonomous mechanism of resistance to sorafenib in HCC as well as inhibiting angiogenesis and reprogramming the immune microenvironment to promote anti-tumor immunity.

## Materials and Methods

### Cells and Materials

We used the murine HCC cell line HCA-1 and HCC1 derived from *Mst1*^−/−^*Mst2*^F/−^ mice and the human HCC cell lines Hep3B, JHH-7, SK-Hep-1, HLF, SNU-449 and SNU-423 (purchased from ATCC, Manassas, VA). Hep3B cells were stably transduced with secreatable Gaussia luciferase (Gluc) gene by using a retroviral vector provided by Dr. Bakhos Tannous, Massachusetts General Hospital for *in vivo* xenograft studies. Hep3B cells were maintained in MEM-alpha medium, HLF and JHH-7 cells were maintained in DMEM/F12 medium, HCC1, SNU-449, SNU-423 and SK-Hep-1 were maintained in RPMI medium. All media were supplemented with 10% fetal bovine serum and were purchased from Invitrogen (Carlsbad, CA). The CTCE peptide (KGVSLSYRCRYSLSVGK) and scrambled peptide (SC) (LYSVKRSGCGSRKVSYL) were synthesized and purified (95% purity) by Kelowna International Scientific Inc. (Taipei, Taiwan). siRNA against ERK1/2 and CRAF and control siRNA were purchased from Dharmacon (Lafayette, CO). Sorafenib, AZD6244 and PLX4720 were obtained from MGH Pharmacy or purchased from Selleck Chemicals (Houston, TX). Dimethyl sulfoxide (DMSO), cholesterol, TPGS and coumarin 6 (C6) were purchased from Sigma-Aldrich (St. Louis, MO). Ethanol was obtained from Alfa Aesar (Ward Hill, MA). 1,2-Dioleoyl-sn-glycero-3-phosphocholine (DOPC) was purchased from Avanti Polar Lipids (Alabaster, AL). PLGA (50/50, inherent viscosity: 0.17 dl/g) was purchased from Green Square Materials Incorporation (Taoyuan, Taiwan). To evaluate NF-kB activity, HCC cells were seeded in 96-well plates and transfected with NF-kB-driven luciferase reporter construct from Agilent technologies (Austin, TX).

### Preparation of nanoparticles

Nanoparticles were prepared via single-step nanoprecipitation as previously described with modifications. Briefly, 0.75 mg of PLGA, 0.15 mg of drug cocktail, 0.375 mg of TPGS, 0.04 mg of DOPC, 0.04 mg cholesterol and 0.08 mg DSPE-PEG(2000)-maleimide in 40 μl of DMSO were mixed as the oil phase and each time 10 μl of oil phase was added to 280 μl of deionized water dropwise under gentle stirring. The NPs were self-assembled with continuous stirring for 30 minutes at room temperature. The ratio of the volumes of the oil and water phases was fixed at 1/7 (v/v) throughout all of the experiments. For peptide conjugation, peptide was reduced using immobilized TCEP disulfide reducing gel (Thermo Scientific) according to manufacturer’s recommendations. Twenty-five μl of 1 mg/ml of CTCE-9908 peptides or scramble peptides were added into the emulsion where it reacted with DSPE-PEG(2000)-maleimide. Four hours later, the unreacted maleimide groups were quenched by adding l-cysteine. The solution was centrifuged at 16,220 rpm for 30 min at 25 °C to collect the NPs, and the NPs were then resuspended in water in the volume equal to the emulsion for characterization. The same procedure was used to synthesize C6-loaded NPs.

### Characterization of nanoparticles

The size and morphology of the NPs were characterized by transmission electron microscopy (TEM; H-7500, Hitachi High-Tech, Tokyo, Japan). The NPs were stained on dried formvar-coated 100-mesh copper grids at room temperature. All grids were further dried again for two days before imaging. The particle size and surface charge were examined using a Zetasizer (3000HS, Malvern Instruments Ltd., Worcestershire, UK) at room temperature.

### *In vivo* HCC models

For orthotopic human HCC model, Gluc-Hep3B cells were orthotopically implanted in livers of 7–8-week-old male nude mice in a 10 μl Matrigel solution. HCC tumors in *Mst1*^–/–^*Mst2*^F/–^ transgenic mice were induced by i.v. injection of Cre-adenoviruses. Male C3H mice were administration with carbon tetrachloride (CCl4) (16% [v/v] in olive oil, 100-ml gavage, 3 times per week) in combination with 5% ethanol in drinking water for 24 weeks to create a chemically induced murine HCC model. For murine orthotopic HCC model, HCA-1 cells were orthotopically implanted in the liver of 7- to 8-week-old male mice in a 100 μl Matrigel solution.

All experiments were performed in accordance with relevant guidelines and regulations. All animals received humane care, in compliance with the “Guide for the Care and Use of Laboratory Animals” published by the National Academy of Sciences, and all study procedures and protocols were approved by the Animal Research Committee of Massachusetts General Hospital and National Tsing-Hua University. Free-form sorafenib was administered daily by gavage at a dose of 50 mg/kg body weight to mice with established tumors. Free-form AZD6244 was administered twice daily by gavage at a dose of 25 mg/kg body weight. For *in vivo* gene silencing, siRNA in LPD formulations were intravenously injected in orthotopic HCC-bearing mice daily at a dose of 1.2 mg of siRNA/kg for 4 days. Tumors were collected 2 hr after the last injection.

### *In vitro* cellular uptake

C6 was formulated in the NPs as a tracer molecule, with a final weight ratio of C6 to PLGA as 1/150. HCA-1, Mahlavu or Hep3B cells (10,000 cells per well) were seeded in a 12-well plate (Costar, IL, USA) and incubated for 12 hours. The cells were then treated with different formulations containing C6 at 37 °C for 4 hours. The cells were washed with PBS, fixed with 4% paraformaldehyde (PFA) for 10 minutes and counterstained with mounting solution (4′,6-diamidino-2-phenylindole or DAPI, Vector Laboratories, Burlingame, CA). The cellular uptake of C6-loaded NPs was examined and quantified using a confocal microscope (LSM-780, Carl Zeiss, Germany).

To perform a CTCE-9908 peptide competitive assay, cells were prepared as previously described. Before treatment with C6-loaded CTCE-NPs, the cells were treated with free CTCE-9908 peptide at a final concentration of 0 μg/ml, 20 μg/ml, or 50 μg/ml for 10 minutes. The cellular uptake of C6-loaded NPs was examined and quantified using a confocal microscope.

### Cell viability assays

Cell viability was assessed using the MTT assay. Cells (10,000 cells per well) were seeded into 96-well plates, allowed to adhere overnight, and exposed to a range of drug concentrations. After 48 or 72 hr, 10 μl of 5 mg/ml MTT dissolved in PBS was added to each well and incubated for 3 hr in 37 °C. The medium was aspirated, and 100 μl of DMSO was added to each well. Absorbance was read at 570 nm.

To analyze the effect of downregulation of CRAF or ERK on *in vitro* cytotoxic effects of sorafenib, cells were transfected with siRNA using Lipofectamine 2000 reagent (Invitrogen). Twelve hours after transfection of siRNA (50 nM), media was replaced with fresh media. The trasfected cells were treated with sorafenib 24 hr after transfection. After 24 hr of sorafenib (2 μM) exposure, apoptosis was detected by staining the cells with Annexin V and propidium iodide solution followed by flow cytometry analysis. In addition, cell viability was assessed using the MTT assay 48 hours after sorafenib treatment.

### Western blot analysis

Cells were lysed in lysis buffer RIPA for 30 min on ice and the supernatant was collected after centrifugation at 12,000 rpm. Cell lysate were separated on a 10% acrylamide gel and transferred to a PVDF membrane. Membranes were blocked for 1 hr in 5% skim milk and then incubated overnight with polyclonal antibodies against p-CRAF, CRAF, p-ERK, ERK, p-p38, p38, p-AKT, AKT, p-IκBα, Bim, p-Bim (Cell Signaling, Danvers, MA) or β-actin (Sigma-Aldrich, St. Louis, MO).

### Immunoprecipitation

HCC1 and Hep3B cell immunoprecipitation was performed using extracts prepared in lysis buffer [0.5% Nonidet P-40, 100 mM NaCl, 10 mM EDTA, 20 mM Tris-HCl (pH 8.0), containing 1 g/ml leu-peptin, 1 g/ml aprotinin, 1 g/ml pepstatin, 0.5 mM dithio-threitol, 0.2 mM sodium vanadate, 100 nM microcysteine]. The extracts were sonicated on ice and clarified by centrifugation at 10,000 rpm for 15 min. BRAF and CRAF was co-immunoprecipitated using the anti-BRAF monoclonal antibody prebound to Protein G beads for Hep3B. CRAF was co-immunoprecipitated using the anti-BRAF monoclonal antibody (Cell Signaling, Danvers, MA) prebound to sepharose A beads for HCC1 cells. Immobilized immune complexes were washed three times, eluted in sample buffer, resolved by SDS-PAGE (10% gels), and transferred to nitrocellulose for immunoblot analysis. Membranes were blocked in 5% milk diluted in PBS for 1 hr, after which they were incubated with BRAF or CRAF primary antibodies diluted in 5% milk/PBS overnight at 4 °C. Membranes were washed in PBST (PBS with 0.1% Tween-20) three times and then incubated for 1 hr with a secondary antibody. Membranes were washed four times and then developed by an enhanced chemiluminescence system according to the manufacturer’s instructions (PerkinElmer).

### Assessment of Apoptosis by TUNEL Staining

Frozen sections of Hep3B tumors were stained by using TACS^TM^ TdT Kit (R&D Systems, Minneapolis, MN) according to the manufacturer’s recommendations. The apoptotic cells were counted in four randomly selected visual fields for each sample. The apoptotic index was calculated as the fraction of apoptotic nuclei.

### Immunofluorescence analyses of HCC tissue

Frozen tumor sections (7–8 μm thick) were immunostained with primary antibodies against CRAF, p-ERK, vWF (Dako, Denmark), PD-L1 (Abcam Inc., Cambridge, MA) Granzyme B (Abcam Inc., Cambridge, MA) and CD8a (BD Biosciences, San Jose, CA) and further stained with fluorescent secondary antibodies. The sections were counterstained with DAPI (Vector Laboratories, Burlingame, CA). Samples were imaged by using Zeiss LSM 780 confocal microscope and quantified using 5–10 randomly selected fields per sample.

### Preparation of PEGylated LPD nanoparticles

Cationic liposomes composed of DOTAP and cholesterol (1:1 molar ratio) were prepared by thin film hydration followed by membrane extrusion to reduce the particle size. To prepare LPD, 18 μl of protamine (2 mg/mL), 140 μl of deionized water, and 24 μl of a mixture of siRNA and calf thymus DNA (2 mg/mL) were mixed and kept at room temperature for 10 min before adding 120 μl of cationic liposome (10 mM). LPD stand at room temperature for 10 min before the addition of DSPE-PEG. LPD was then mixed with 40 μl of DSPE-PEG (17 mg/mL) and kept at 50–60 °C for 10 min.

### Patients and HCC tissue

HCC tissue was obtained through tumor biopsies of patients who had undergone liver resection at the department of Surgery at the Far Eastern Memorial Hospital, Taiwan and at Fundeni Clinical Institute, Bucharest, Romania. All experiments were performed in accordance with relevant guidelines and regulations and all study procedures and protocols were approved by the Institutional Review Boards of Far Eastern Memorial Hospital (FEMH No. 104192 F) and Fundeni Clinical Institute. Written informed consent was obtained from all patients prior to the operation.

### Statistical analysis

All statistical analyses were performed by student *t*-test. Data were considered statistically significant when *p* value was less than 0.05.

## Additional Information

**How to cite this article:** Chen, Y. *et al*. Overcoming sorafenib evasion in hepatocellular carcinoma using CXCR4-targeted nanoparticles to co-deliver MEK-inhibitors. *Sci. Rep.* **7**, 44123; doi: 10.1038/srep44123 (2017).

**Publisher's note:** Springer Nature remains neutral with regard to jurisdictional claims in published maps and institutional affiliations.

## Supplementary Material

Supplementary Information
