# Supplementary material for: Overcoming sorafenib evasion in hepatocellular carcinoma using CXCR4-targeted nanoparticles to co-deliver MEK-inhibitors
Source: Sci Rep. 2017 Mar 9;7:44123. doi: 10.1038/srep44123 (PMC5343435; doi:10.1038/srep44123)
Supplement: Supplementary Information [file srep44123-s1.doc]

Overcoming sorafenib evasion in hepatocellular carcinoma using CXCR4-targeted nanoparticles to co-deliver MEK-inhibitors

Yunching Chen^1,2,†^,*, Ya-Chi Liu^2,†^, Yun-Chieh Sung^2,†^, Rakesh R. Ramjiawan^1,3^, Ts-Ting Lin^2^, Chih-Chun Chang^2^, Kuo-Shyang Jeng^4^, Chiung-Fang Chang^4^, Chun-Hung Liu^2^, Dong-Yu Gao^2^, Fu-Fei Hsu^5^, Annique M. Duyverman^1,6^, Shuji Kitahara^1^, Peigen Huang^1^, Simona Dima^7^, Irinel Popescu^7^, Keith T. Flaherty^8^, Andrew X. Zhu^8^, Nabeel Bardeesy^8^, Rakesh K. Jain^1^, Cyril H. Benes^8^, and Dan G. Duda^1^,*

From the: ^1^Steele Laboratories for Tumor Biology, Department of Radiation Oncology, Massachusetts General Hospital and Harvard Medical School, Boston, USA; ^2^Institute of Biomedical Engineering, National Tsing Hua University, Hsinchu, Taiwan; ^3^Angiogenesis Laboratory, Department of Medical Oncology, VU University Medical Center, Amsterdam, The Netherlands; ^4^Department of Surgery, Far Eastern Memorial Hospital, New Taipei City, Taiwan; ^5^Institute of Biomedical Sciences, Academia Sinica, Taipei, Taiwan; ^6^University Medical Center, Utrecht, The Netherlands; ^7^Dan Setlacec Center of General Surgery and Liver Transplantation, Fundeni Clinical Institute, Bucharest, Romania. ^8^Department of Medicine, Massachusetts General Hospital and Harvard Medical School, Boston, USA.

**Short Title: Sorafenib nanodelivery to overcome HCC evasion**

*Corresponding authors: Dan G. Duda, DMD, PhD, 13th Street, CNY 3.407, Charlestown, MA 02129; phone: (617) 726-4648; fax: (617) 726-1962; email: gduda@partners.org, or Yunching Chen, PhD, Institute of Biomedical Engineering, National Tsing Hua University, Hsinchu 30013, Taiwan, ROC; phone: 886-3-571-5131 ext: 35503; email: yunching@mx.nthu.edu.tw

^†^These authors contributed equally to this work.

**Supplementary Materials and Methods**

**Luciferase reporter assay**

We used the luciferase reporter assay to detect NF-κΒ activity after treatment of sorafenib and AZD6244 using a NF-κΒ luciferase reporter vector obtained from Agilent technologies (Austin, TX). HCA1 and Hep3B cells were seeded in 96 well plates. Twelve hours after cell seeding, cells were transfected with NF-κΒ luciferase reporter vector (100ng in 100μl medium) with lipofectamine 2000 (Thermo Fisher Scientific). Twenty-four hours after transfection, cells were treated with different concentration of sorafenib and AZD6244. The luciferase activity was measured by Dual Luciferase assay kit (Promega, USA) after 24hr of drug exposure.

**Supplementary Table S1. Effect of sorafenib on activation of the signal transduction pathways in HCC.** N/A, Not available.


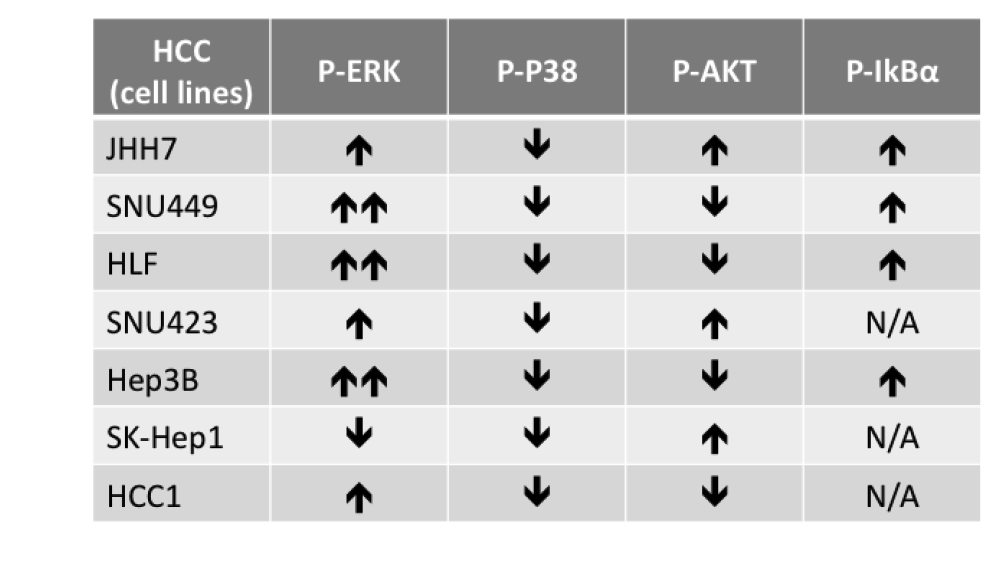


**Supplementary Table S2. Toxicity profile of sorafenib and AZD6244-loaded CTCE NPs.** Sera of C3H mice were collected 24 hr after IV injections of sorafenib and AZD6244-loaded CTCE NPs for evaluation of liver enzyme levels. Abbreviations: ALT, alanine aminotransferase; AST, aspartate aminotransferase; ALP, alkaline phosphatase; γ-GT, γ-Glutamyltransferase. Data are shown as mean values ± SEM.

**
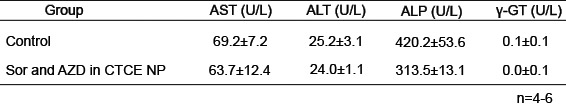
**


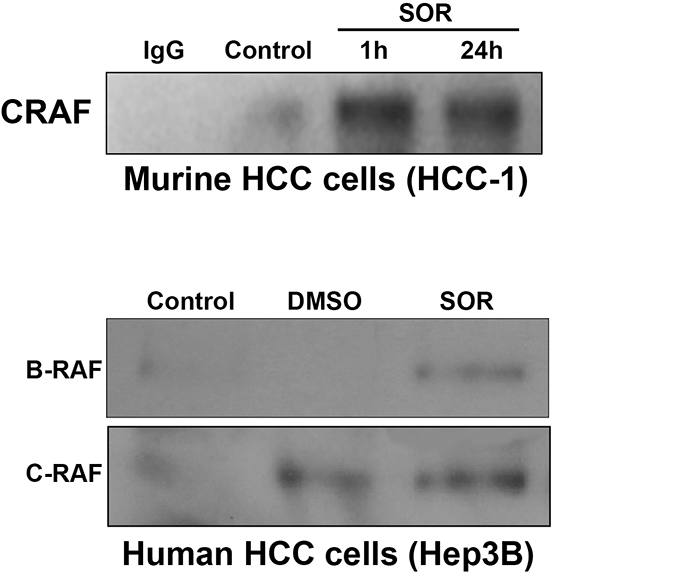


**Supplementary Fig. S1. Transactivation of RAF dimers occurred after treatment of sorafenib.** Murine HCC cells (HCC-1 cells) were treated with either vehicle or 2µM sorafenib for 1 hr and 24 hr. Activation in the context of the heterodimer BRAF/CRAF occurs after treatment of sorafenib.


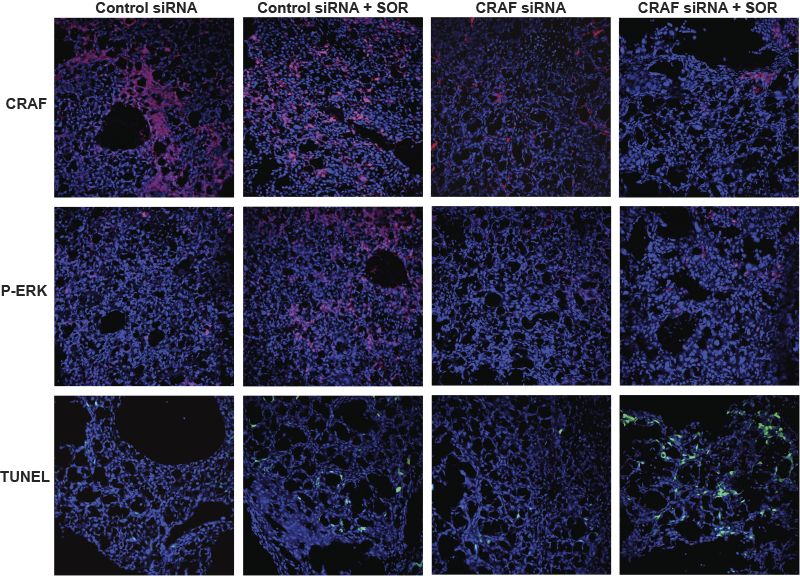


**Supplementary Fig. S2. Paradoxical activation of ERK promotes sorafenib resistance in *BRAF*-wild type HCC cells *in vivo.*** Combination of sorafenib with CRAF siRNA encapsulated in liposome-based nanoparticles silenced CRAF expression, inhibited ERK activation and increased cell apoptosis in orthotopic Hep3B xenografts in nude mice.

**Supplementary Fig. S3. CTCE NPs showed the enhanced cellular uptake in HCC cells.** **a,** Various HCC cells (Mahlavu, Hep3B and HCA-1 cells) were treated with C6-loaded CTCE-NPs for 4 hr. The cellular uptake of NPs was imaged and quantified with a Zeiss LSM 780 confocal microscope. **b**, The uptake of CTCE-NPs was competitively inhibited by addition of free CTCE-9908 peptides in a dose-dependent manner. Cells were treated with CTCE peptides prior to CTCE-NPs and analyzed for fluorescence signal by the confocal microscopy.

**
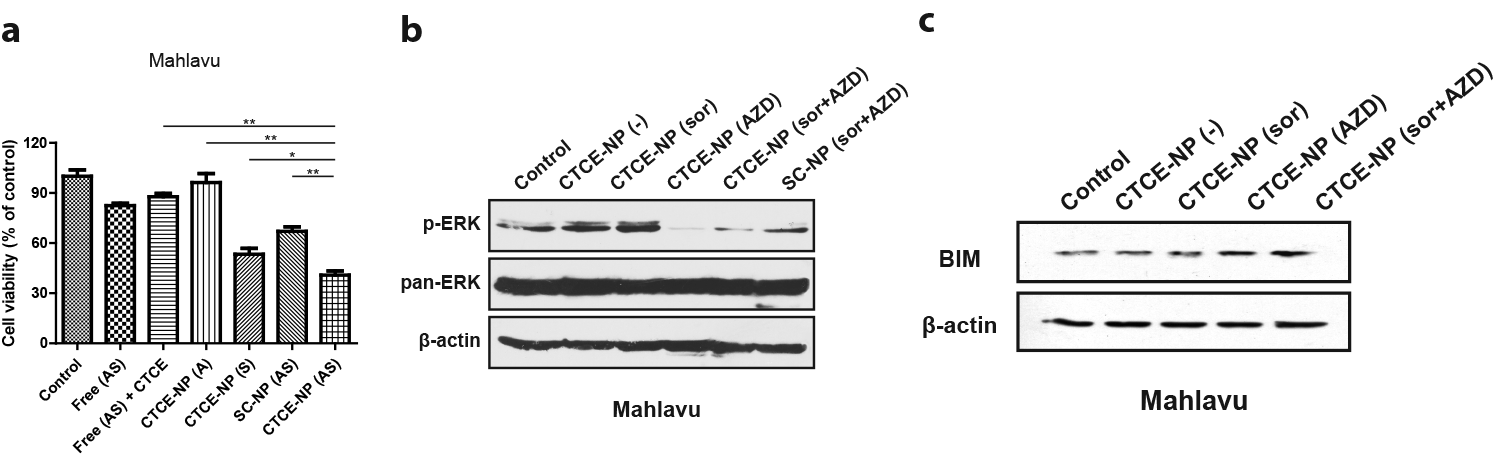
**

**Supplementary Fig. S4. CTCE-NPs loaded with sorafenib and the MEK inhibitor AZD6244 exerted potent cytotoxic effects, prevented the paradoxical activation of ERK and increased the expression of Bim on human HCC cells. a,** The cytotoxicity of sorafenib or AZD6244 in different formulations to Mahlavu cells was measured using the MTT assay (n=4-6). **b**, CTCE-NPs co-delivering sorafenib and AZD6244 prevented the effect of sorafenib on paradoxical activation of ERK in Mahlavu cells. **c**, CTCE-NPs loaded with sorafenib and AZD6244 upregulated expression of Bim in Mahlavu cells. The data are shown as mean values ± S.E.M., *p<0.05, **p<0.01.

**
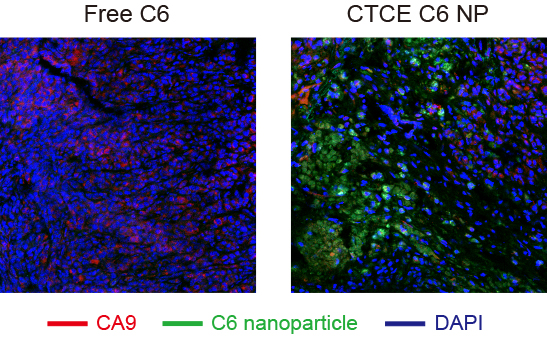
**

**Supplementary Fig. S5. Heterogeneous uptake and distribution of nanoparticles in HCC**. The cells that took up the NPs were not in the hypoxic regions in the tumor tissues analyzed.

**
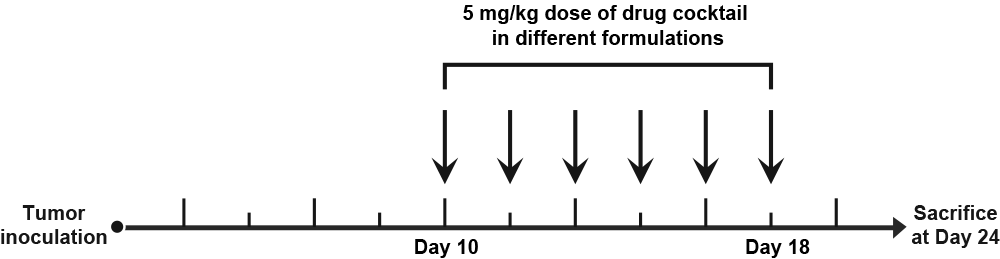
**

**Supplementary Fig. S6. Treatment schedule of sorafenib and AZD6244 in different formulations in the orthotopic HCC model.**


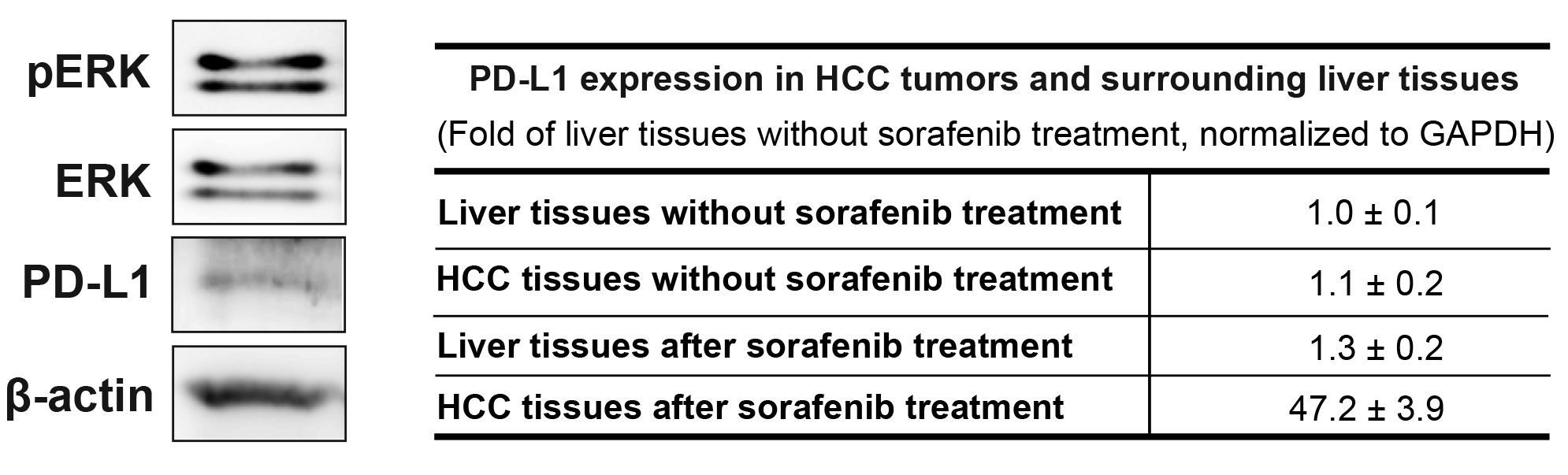


**Supplementary Fig. S7. Activation of ERK and increased expression of PD-L1 in recurrent human HCC after sorafenib treatment (n=2).**

**
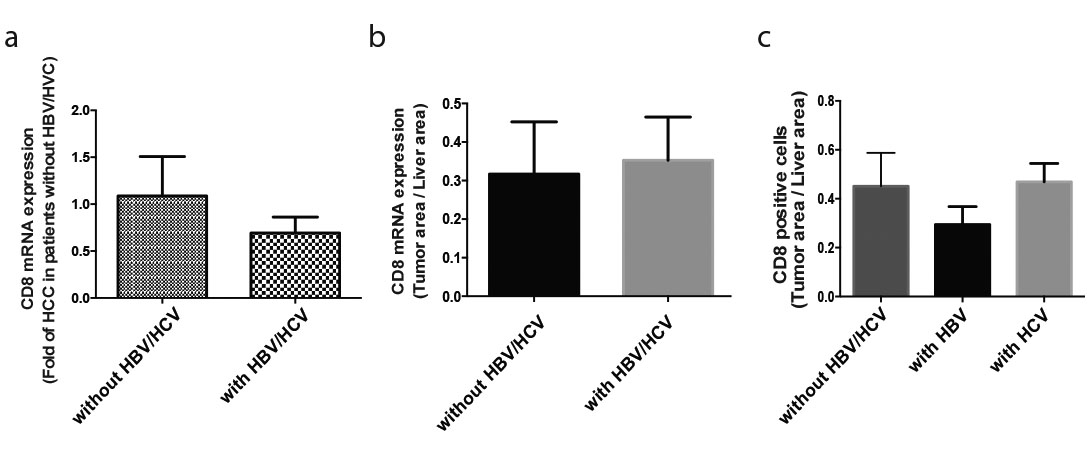
**

**Supplementary Fig. S8. Lymphocyte infiltration in human HCC. a,** Viral infection (HBV/HCV) does not appear to significantly affect the tumor infiltration of CD8+ T-lymphocytes. **b**, Expression of CD8 in tumor tissue was lower than in the liver tissues, suggesting a reduced infiltration by CD8+ T-lymphocytes into the tumors. **c**, Tumor-infiltrating CD8+ T cells were analyzed by immunohistochemistry. The infiltration of CD8+ T cells in tumor tissues was lower than in liver tissues both in patients with and without HBV/HCV infection.


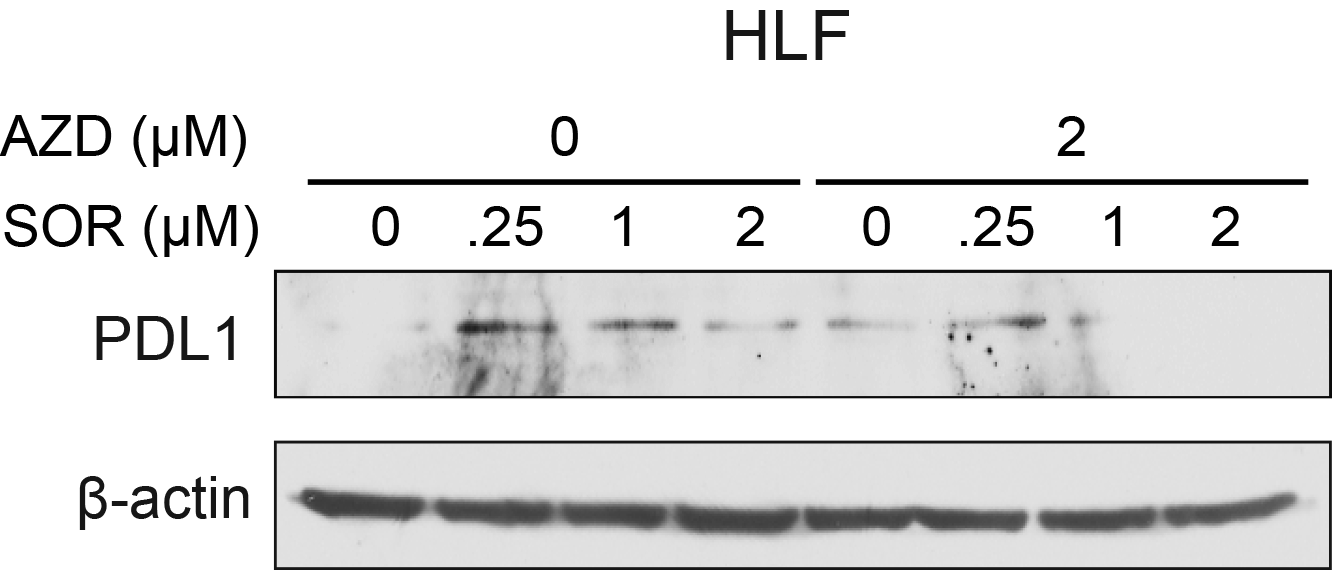


**Supplementary Fig. S9. Treatment of sorafenib at low doses increased the expression of PD-L1 in the human HCC cell line HLF, consistent with their paradoxical activation of ERK, while inhibition of MEK with AZD6244 prevented the effects of sorafenib.**

**
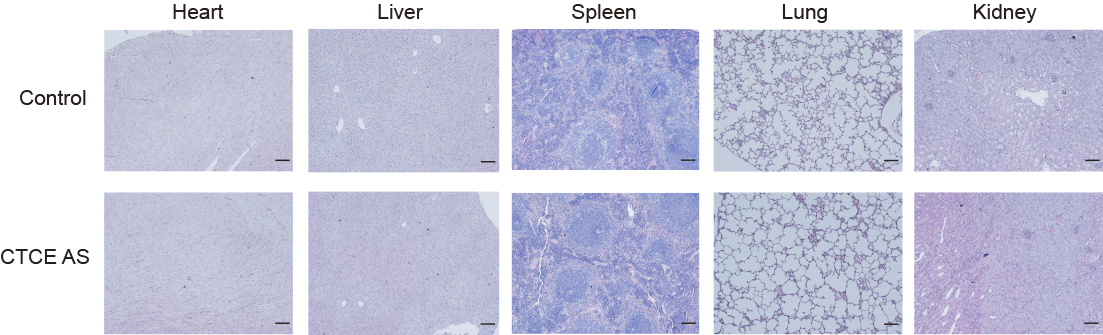
**

**Supplementary Fig. 10. H&E staining revealed no histological change in the various organs including lungs, livers, spleens, kidneys and hearts of C3H mice 24 hours after treatment with sorafenib and AZD6244 in CTCE-NPs (scale bar = 100 μm).**
